# Perioperative management of open fractures in the lower limb

**DOI:** 10.1177/17504589211012150

**Published:** 2021-07-02

**Authors:** Bryan Loh, Jiang An Lim, Matthew Seah, Wasim Khan

**Affiliations:** 1 Department of Trauma and Orthopaedics, Addenbrookes Major Trauma Unit, Cambridge University Hospitals, Cambridge, UK; 2 School of Clinical Medicine, University of Cambridge, Cambridge, UK

**Keywords:** Perioperative management / Trauma and orthopaedic surgery / Plastic surgery / Open fractures / Lower limb fractures

## Abstract

An open fracture is a fracture which communicates with the external environment through a wound in the skin. Severe open fractures are managed by both orthopaedic and plastic surgeons to address injuries in both the bone and soft tissue. This review outlines the management of open fractures in the lower limb from the initial patient presentation to operative management (including debridement, skeletal fixation, definitive soft tissue coverage) according to the standards jointly published by the British Orthopaedic Association (BOA) and the British Association of Plastic, Reconstructive and Aesthetic Surgeons (BAPRAS). Additionally, the decision-making between limb salvage or amputation will be explored. Finally, this review will discuss the patient’s postoperative care including wound care and management of potential complications that may arise such as infection, flap failure and fracture non-union.

**Provenance and Peer review:** Unsolicited contribution; Peer reviewed; Accepted for publication 3 April 2021.

## Introduction

An open fracture is a fracture which communicates with the external environment through a wound in the skin (Mosheiff 2018). Open fractures are usually high energy traumatic injuries which may arise from sports, road traffic accidents or blast injuries in conflict zones (Jordan et al 2014). Open fractures are at a greater risk of infections, delayed union, non-union and delayed return to function compared to closed fractures particularly when they are not managed appropriately (Mosheiff 2018, Papakostidis et al 2011). The management of open fractures involves the removal of contaminants and non-viable tissue, followed by closure such that they are converted to closed fractures when the fracture is stabilised (Nanchahal et al 2009).

In the pre-hospital setting, members of the ambulance service team will identify the severity of the open fracture based on the pattern of fracture and soft tissue injury (NICE 2016a). Features of severe open fractures include significant fragmentation of the bone (comminution or segmentation), bone loss and skin loss such that tension-free closure is not possible following wound excision or injury to one of the major arteries of the leg. The complete criteria may be found in the guidelines jointly published by the British Orthopaedic Association (BOA) and the British Association of Plastic, Reconstructive and Aesthetic Surgeons (BAPRAS) (Nanchahal et al 2009). Severe open fractures would prompt direct transfer to a specialist centre with Orthoplastic care so that the injury can be managed by both orthopaedic and plastic surgeons (Nanchahal et al 2009). Where geographical barriers exist and the patient is in a critical condition, transfer to the specialist centre may be indirect via the nearest local emergency department so that intermediate care may be provided (NICE 2016b).

Although the standards published by BOA and BAPRAS only regard the management of open fractures in the lower limb, the management of open fractures in the hand, wrist or digit follows similar principles (BOAST 2020). This review will discuss the BOA and BAPRAS guidance on the management of lower limb open fractures in the context of perioperative care.

## Preoperative management

### Primary survey (Advanced Trauma Life Support) and examination of the injured limb

The initial approach to the injured patient with an open fracture should follow Advanced Trauma Life Support (ATLS) principles where life-threatening problems are identified and managed in a logical, hierarchical sequence such that the most imminent threat to life is addressed first (Nanchahal et al 2009). This can be undertaken by anyone who is trained in ATLS (ATLS 2018). Firstly, the patient’s cervical spine should be stabilised, followed by the assessment of their airway, breathing, circulation, disability (ie: assessment of their neurological state) and finally exposure of the patient which may be necessary to carry out a full examination – this follows the mnemonic of ABCDE (airway, breathing, circulation, disability and exposure) (ATLS 2018).

The injured limb should be examined systematically and its neurovascular status documented before and after any manipulation (Nanchahal et al 2009). Should compartment syndrome or arterial injuries be suspected, management should be by the respective BOA Standards for Trauma and Orthopaedics (BOAST) guidelines (BOAST 2020) (see Table 1). The open fracture wound should also be photographed (Nanchahal et al 2009).

**Table 1:** Clinical signs and management of compartment syndrome and arterial injury (BOAST 2020)

	Signs	Management
Compartment syndrome	• Pain out of proportion to the associated injury • Pain on passive movement of the muscles of the involved components • May require compartment pressure measurements if clinical symptoms and signs cannot be readily identified (eg: unconscious patient or patient has nerve block)	• All circumferential dressings released to skin and the limb elevated to heart level • Maintain a normal blood pressure • Surgical decompression if symptoms persist after 30min or if absolute compartment pressure is greater than 40mmHg
Arterial injury	• Altered sensation, continued blood loss, expanding haematoma and absent pulse(s)	• A devascularised limb requires urgent surgical exploration to attempt revascularisation • In some cases, an amputation may be performed

In 2016, NICE recommended intravenous morphine as the first-line analgesic for major trauma (including open fractures) and to adjust the dose as needed to achieve adequate pain relief. Outside of the sterile theatre environment, gross contaminants may be removed but exploration and irrigation of the wound are not recommended as they increase the risk of infection (Nanchahal et al 2009). A sterile saline dressing may be applied to the wound and sealed with an adhesive film for physical protection and from environmental contamination (Nanchahal et al 2009). The injured limb should then be re-aligned and splinted to ensure stability while the patient is in transit, otherwise, motion at the fracture site will add to the initial damage (Gueorguiev-Rüegg & Stoddart 2018).

### Prophylactic antibiotics

Intravenous prophylactic antibiotics should be administered within 3h of injury or as soon as possible and the choice of antibiotics should consider the local trust guidelines and the patient’s allergy status (Nanchahal et al 2009). BOA and BAPRAS guidelines propose intravenous co-amoxiclav (1.2g) or cefuroxime (1.5g) 8h, but if the patient is allergic to penicillin then clindamycin (600mg) should be given (Nanchahal et al 2009). The patient may also require tetanus prophylaxis depending on their vaccination history (Nanchahal et al 2009).

### Imaging

When patients receive a trauma computed tomography (CT) scan to visualise the damage to the bones non-invasively, there should be protocols in place which may include a head-to-toe ‘scanogram’ which can then be used with clinical correlation to direct further specific limb sequences with or without CT angiography and avoid missing any findings and delays from re-scanning (NICE 2016b). Imaging is only useful if it may alter the approach to intervention; therefore it should only take place at a centre where an appropriate follow-up intervention is available; but if it were to be taken for use later, it should not delay transfer to the described centre (ATLS 2018).

### Classification and scoring of open fractures

Classification systems aid the description of the open fracture injury, which may influence management or predict prognosis (Jordan et al 2014).

One example is the Gustilo-Anderson system which is best applied after wound debridement. Type I-III injuries were first described by Gustilo & Anderson (1976) and the Type III injuries were further subdivided by Gustilo et al (1984) (see Table 2).

**Table 2:** The Gustilo-Anderson classification system (Gustilo et al 1984)

Gustilo-Anderson grade	Description
Type I	Wound <1cm long, clean and limited soft tissue damage
Type II	Wound >1cm but <10cm long, without substantial soft tissue damage
Type IIIa	Enough soft tissue coverage for primary closure
Type IIIb	Not enough soft tissue coverage, necessitating a skin graft, local or free flap
Type IIIc	Associated with an arterial injury requiring repair, irrespective of the degree of soft tissue damage

There are also scoring systems designed to aid a surgeon in contemplating whether to amputate or salvage a severely injured lower limb, such as the Mangled Extremity Severity Score (MESS) (Johansen et al 1990) (see Table 3), Nerve Injury, Ischaemia, Soft tissue injury, Skeletal injury, Shock and Age of patient (NISSSA) score (McNamara et al 1994) and Limb Salvage Index (Russell et al 1991).

**Table 3:** The MESS system (Johansen et al 1990)

	Score	Description
Skeletal soft tissue injury	1	Low energy
	2	Moderate energy
	3	High energy
	4	Very high energy
Limb ischaemia, double the score if ischaemia is greater than 6h	1	Pulse reduced or absent but normal perfusion
	2	Pulseless, paresthesia, slow capillary refill
	3	Cold, paralysed, numb
Shock	1	Systolic blood pressure always > 90mmHg
	2	Transient hypotension
	3	Consistent hypotension
Age	1	<30 years old
	2	30 to 50 years old
	3	>50 years old

## Operative management

Where the patient has mental capacity, their consent for any intervention will have to be sought (GMC 2008). If patients were unable to consent (eg: they are unconscious) in an emergency setting, healthcare professionals can treat the patient without their consent if the treatment is immediately necessary to save their life or to prevent a serious deterioration of their condition (GMC 2008).

A National Confidential Enquiry into Patient Outcome and Death (NCEPOD) classification is assigned by the surgeon to define the priority for surgical intervention, whether it is immediate (decision to operate within minutes), urgent (decision to operate within hours), expedited (decision to operate within days) or elective (timing of operation to suit patient, hospital and staff) (Alleway 2004). Where surgical intervention is neither immediate nor urgent, the anaesthetist should take a history from the patient (eg: allergies, smoking status) which may alter the approach to their operative and postoperative care (Verma et al 2010).

### Formal debridement

The removal of contaminants and non-viable tissue, which may eventually become a focus of infection, from a wound is known as debridement (Nanchahal et al 2009). Debridement is described as ‘formal’ when performed in theatre.

Debridement should only be immediate after preoperative management if the wound is expected to be contaminated with a high bacterial load such as sewage or dirt, if compartment syndrome is present, if arterial supply to the limb is compromised or if the patient has multiple injuries (Nanchahal et al 2009). Otherwise, for isolated high energy open fractures, debridement should be done within 12h and for low energy open fractures, within 24h (Nanchahal et al 2009).

The patient is first anaesthetised (Nanchahal et al 2009). General anaesthesia may be preferred over a regional nerve block if the patient’s limb is at high risk of compartment syndrome, as the clinical signs associated with the evolvement of compartment syndrome would be masked by the latter (BOAST 2020). The injured limb is then cleaned with a soapy solution (Nanchahal et al 2009). The surgeon may choose to apply a tourniquet to reduce bleeding which may obscure the underlying anatomy (Nanchahal et al 2009). However, a degree of bleeding can be useful as a sign of tissue viability as visibility can still be maintained with intermittent irrigation and suction (Nanchahal et al 2009). Other considerations include the risk of ischaemia associated with tourniquet use and subsequent reperfusion injury following release (Halladin et al 2014).

Debridement should be done systematically, layer by layer from superficial to deep and from compartment to compartment (Nanchahal et al 2009). As part of the World Health Organization surgical safety checklist, any important information regarding the patient and the operation should be discussed in the team brief (WHO 2008).

Access to deeper structures for debridement may be achieved by extending the wound along fasciotomy lines (Nanchahal et al 2009). Bone fragments are subjected to the ‘tug test’ and if they separate easily, are removed (Nanchahal et al 2009). A second look procedure may be appropriate 24 to 48h later to re-assess tissue viability and to ensure that all non-viable tissue has been removed (Nanchahal et al 2009). This is followed by lavage, preferably at low pressure and with warm saline to clear surface debris (Nanchahal et al 2009). There is no evidence to show that outcomes are improved by adding soap or antibiotics to the lavage fluid (Anglen 2005).

Following debridement, skeletal fixation and soft tissue reconstruction may be carried out within the same session (Nanchahal et al 2009). If skeletal and soft tissue reconstruction were to take place in a separate session, a vacuum foam dressing or antibiotic bead pouch is applied until then (Nanchahal et al 2009). For Gustilo grade II and III fractures, antibiotics should be prescribed until definitive skin closure or for a maximum of 72h, whichever is shorter (Nanchahal et al 2009).

### Definitive soft tissue reconstruction

Where the wound is too large to be closed directly, soft tissue reconstruction is performed (Simman 2009). The choice of soft tissue reconstruction follows the reconstructive ladder which explores the simplest to the most complex option, starting with skin grafts, local flaps and finally free flaps (Simman 2009) (see Table 4).

**Table 4:** The reconstructive ladder (Simman 2009)

Types of soft tissue reconstruction	Description
Skin graft	• involves the transplantation of the tissue, with or without its blood supply, from the donor site to the recipient wound site • may be full thickness if they contain the epidermis and the entire dermis or split-thickness if they contain the epidermis with a portion of the dermis
Local flap	• involves the transplantation of the tissue, together with its blood supply, from the donor site to the recipient wound site • a local flap is located next to the wound and the skin is left attached on one end to preserve the blood supply while the other end is moved to cover the wound
Free flap	• like a local flap, involves the transplantation of the tissue, together with its blood supply, from the donor site to the recipient site • unlike a local flap, the free flap is obtained distant from the wound and the blood vessels are anastomosed by microsurgery

Studies have shown that if definitive soft tissue reconstruction is achieved earlier, flap survivability improves, infection rates are lower (Qiu et al 2018) and occurrence of osteomyelitis is reduced (Breugem & Strackee 2006). Therefore, current guidelines suggest that it should be achieved as promptly as possible after debridement and certainly within 72h of injury (BOAST 2020). It should be noted that the use of temporary dressings such as vacuum foam dressing does not nullify the negative outcomes associated with delaying soft tissue reconstruction and hence should never be a definitive substitute (Hou et al 2011).

### Skeletal fixation

Skeletal fixation is required to keep the fracture ends in close apposition and stable so that bone healing (osteogenesis) can take place (Sathyendra & Darowish 2013). This stability may be absolute or relative to promote primary and secondary bone healing, respectively (Sathyendra & Darowish 2013).

Another important element that affects fracture healing is the blood supply and this is provided by the soft tissue surrounding the bone (Marsh & Li 1999), such that soft tissue damage can delay fracture union (Rommens & Broos 1992). Consequently, when fixing the fracture, one should attempt to minimise soft tissue and periosteal damage (Davis et al 2015). In open fractures with extensive soft tissue injury, external fixation may be preferred over internal fixation, as it causes less disruption to the soft tissues (Jordan et al 2014). The use of external fixation is usually temporary, with later conversion to internal fixation but in some cases, may be final (ie definitive) (Beltsios et al 2009).

Definitive internal fixation should only be carried out when it can be followed immediately with definitive soft tissue coverage, otherwise, the risk of infection may be increased significantly (Nanchahal et al 2009).

The exact method of fixation is determined by many factors including the degree of soft tissue injury, fracture location, fracture pattern, patient-related factors, the mechanism of injury and the surgeon’s expertise (Mosheiff 2018).

### The decision between limb salvage or primary amputation

When managing a patient with severe limb trauma, the decision for primary amputation should be weighed against the impact of limb salvage and reconstruction (Nanchahal et al 2009). The decision to undertake a primary amputation should be made by two consultant surgeons where possible (Nanchahal et al 2009). Indications for primary amputation may include damage control (amputation as the only means of haemorrhage control and resuscitation) and limb ischaemia (eg: an avascular limb with a warm ischaemic time exceeding 4h) (Nanchahal et al 2009). Where the presenting injury is not as well-defined, the factors to be taken into consideration extend beyond anatomical and functional variables, and patient factors should also be considered (eg: physiological, psychological, social) (Nanchahal et al 2009). Scoring systems like MESS (Johansen et al 1990), NISSSA (McNamara et al 1994) and the Limb Salvage Index (Russell et al 1991) have been developed to aid the surgeon’s decision-making, but these have demonstrated limited benefit (Nanchahal et al 2009).

Where delayed amputation is considered, a multidisciplinary assessment (eg: orthopaedics, plastic surgery, rehabilitation medicine) should be carried out with the patient and their family or carers (Nanchahal et al 2009).

## Postoperative care

Immediately after surgery, the patient is transferred to the post-anaesthesia care unit (PACU) where the patient’s vital signs and airways are monitored by the recovery nurse or ODP as they recover from the effects of anaesthesia (The Royal College of Anaesthetists [RCoA] 2019). Following PACU, patients are usually transferred back to the wards unless there is a life-threatening complication for which they would be transferred to an intensive care unit (RCoA 2019).

The treated limb should be elevated to minimise swelling (Fan and Arraf 2018). Where appropriate, the limb should be immobilised in a cast or splint until the fracture heals (Fan & Arraf 2018). Patients should be assessed for venous thromboembolism (VTE) risk, most commonly with the Department of Health VTE risk assessment tool, as soon as possible after admission and receive VTE prophylaxis if indicated (NICE 2018). Anti-embolism stockings should not be worn over wound areas. In these cases, pharmacological prophylaxis such as low-molecular weight heparin or direct oral anticoagulants may be preferred (NICE 2018). Patients should be informed, either by written or verbal means, of their prognosis and advised on their return to normal activities (Moran 2018, NHS England Patient and Public Participation and Insight Group 2016).

Physical therapy (mobilisation and weight-bearing) should commence as soon as possible to reduce the risk of developing osteopenia, where bone density is lower than normal, and sarcopenia which is the loss of skeletal muscle mass and function (Fan & Arraf 2018). Early mobilisation can reduce the risk of VTE and promote angiogenesis to produce a more favourable environment for osteogenesis (Jordan et al 2014).

### Wound care

While the patient is in the ward, the wound should be kept clean by having the wound cleansed and the dressing changed by medical staff to reduce surgical site infection (NICE 2021). Patients should be taught by the medical staff to monitor the condition of their wound post-operatively (Public Health England 2013).

The monitoring of flaps should include the observation of its colour and physical examination of its capillary refill time, turgor and temperature in comparison to the adjacent skin that is not a flap (Chao & Lamp 2014). Doppler ultrasound may also be used to investigate the blood flow to the flap (Chao & Lamp 2014). Early intervention improves the chance of salvaging a failing flap (Chen et al 2007). Pressure on flaps may compromise its vascular supply; thus, it should be avoided by ensuring that dressings are not too tight (Chao & Lamp 2014). There has been no unified regime for the monitoring of flaps across plastic surgery units in the United Kingdom, although the most common was found to be one hourly observations in the first 24h, one to two-hourly observations in the next 24h, and every four hours in the third 24h period (Jallali et al 2005).

### Patient-dependent factors

Certain patient factors are known to be associated with poorer outcomes. Smoking is associated with a higher rate of surgical site infection, delayed fracture union and non-union (Moghaddam et al 2011). Additionally, patients with older age, diabetes mellitus, hypertension and peripheral vascular disease were found to be associated with increased risk of flap failure or necrosis (Baumeister et al 2003, Las et al 2016). Therefore, it is important to consider patient factors when planning the management of open fractures and establishing a realistic prognosis (Mosheiff 2018). These factors should have been identified at the assessment stage by the anaesthetist (Verma et al 2010).

Patients should also receive proper nutritional support when in the recovery phase which may help with wound healing and reducing infection risk (Jordan et al 2014).

## Complications associated with open fractures

### Infection

Infection may be superficial, occurring in soft tissue, or deep, occurring in the bone (such as osteomyelitis) or in the implant (Kaufman et al 2016). This may be the result of microbes that the fracture wound may have been exposed to at the time of injury, nosocomial microbes acquired in the hospital, failure to remove contaminants, or inadequate debridement of necrotic tissue (Nanchahal et al 2009, Zalavras and Patzakis 2003).

The risk of infection has shown to be greater with higher grades of open fracture (Zalavras & Patzakis 2003) (see Table 5) and delayed wound closure (Lo et al 2007).

**Table 5:** Infection rate for different grades of open fracture (Zalavras & Patzakis 2003)

Fracture grade	Infection rate (%)
Gustilo-Anderson type I	0 to 2
Gustilo-Anderson type II	2 to 10
Gustilo-Anderson type III	10 to 50

While superficial wound infections may be treated with antibiotics and a change in dressing, the management of deep infection necessitates a return to the operating theatre for fracture site investigation, debridement and possible revision of the skeletal fixation (Nanchahal et al 2009). If left unchecked, an infection may complicate into flap failure, fracture non-union or sepsis (Nanchahal et al 2009).

### Non-union

Non-union is the failure of a fracture to heal, which will remain as such without further intervention (Elliott et al 2016). While septic non-union is caused by infection, aseptic non-union is caused by poor blood supply or poor bone stability preventing fracture healing (Elliott et al 2016). Aseptic non-union is divided into hypertrophic and atrophic non-union (Elliott et al 2016). Hypertrophic non-union occurs when poor stability at the fracture site becomes the limiting factor for fracture healing, while in atrophic non-union, poor vascular supply or insufficient mobilisation results in an impaired biological healing response (Elliott et al 2016). Further surgery may be required to revise the skeletal fixation, remove non-viable fracture ends or insert a bone graft to make up for the bone loss (Nanchahal et al 2009).

### Compartment syndrome

Acute compartment syndrome refers to ischaemia of the muscles and nerves within a fascial compartment due to raised intra-compartmental pressure (ICP) which reduces tissue perfusion (Nanchahal et al 2009). This is a surgical emergency and a high index of suspicion must be maintained so that this is promptly diagnosed and treated by fasciotomy, where the fascia is cut to relieve the raised ICP (Nanchahal et al 2009). The key feature of compartment syndrome in the conscious patient is severe pain, out of proportion to the injury and aggravated by passive muscle stretch (BOAST 2020). In an obtunded patient (eg: due to delirium, intoxication, sedation) or when there are distracting injuries, there may be a role for ICP monitoring or serial ICP measurements of the affected limb (BOAST 2020).

## Conclusion

This review summarises the salient points in the perioperative management of open fractures in the lower limb per current guidelines from BOAST and BAPRAS. We have not discussed areas of ongoing debate where findings have been inconclusive (Jordan et al 2014). These guidelines are likely to be provisional, as they may evolve continually to keep up with new evidence. Additionally, the current guidelines only focus on open fractures in the lower limb. Although the management of open fractures in other parts of the body (eg: hand, wrist or digit) follows a similar process (BOAST 2020), there are currently no specific guidelines and this may change in the future.

## Key phrases

1. Open fractures should be managed by both orthopaedic and plastic surgeons to address the bone and soft tissue injuries.
2. The management of open fractures involves early wound debridement, and closure such that they are converted to closed fractures when the fracture is stabilised.
3. Open fracture classification and scoring systems aid the description of the open fracture injury, which may influence management or predict prognosis.
4. Complications associated with open fractures include infection, flap failure and fracture non-union.

*No competing interests declared.*
